# Association of Data Integration Technologies With Intensive Care Clinician Performance: A Systematic Review and Meta-analysis

**DOI:** 10.1001/jamanetworkopen.2019.4392

**Published:** 2019-05-24

**Authors:** Ying Ling Lin, Patricia Trbovich, Lauren Kolodzey, Cheri Nickel, Anne-Marie Guerguerian

**Affiliations:** 1 Institute of Biomaterials and Biomedical Engineering, Faculty of Engineering, University of Toronto, Toronto, Ontario, Canada; 2 Department of Critical Care Medicine, The Hospital for Sick Children, Toronto, Ontario, Canada; 3 Institute of Health Policy, Management and Evaluation, University of Toronto, Toronto, Ontario, Canada; 4 Badeau Family Research Chair in Patient Safety and Quality Improvement, North York General Hospital, University of Toronto, Toronto, Ontario, Canada; 5 Hospital Library and Archives, The Hospital for Sick Children, Toronto, Ontario, Canada; 6 Interdepartmental Division of Critical Care Medicine, University of Toronto, Toronto, Ontario, Canada; 7 Neurosciences and Mental Health Program, Research Institute, The Hospital for Sick Children, Toronto, Ontario, Canada

## Abstract

**Importance:**

Sources of data in the intensive care setting are increasing exponentially, but the benefits of displaying multiparametric, high-frequency data are unknown. Decision making may not benefit from this technology if clinicians remain cognitively overburdened by poorly designed data integration and visualization technologies (DIVTs).

**Objective:**

To systematically review and summarize the published evidence on the association of user-centered DIVTs with intensive care clinician performance.

**Data Sources:**

MEDLINE, Embase, Cochrane Central Register of Controlled Trials, PsycINFO, and Web of Science were searched in May 2014 and January 2018.

**Study Selection:**

Studies had 3 requirements: (1) the study tested a viable DIVT, (2) participants involved were intensive care clinicians, and (3) the study reported quantitative results associated with decision making in an intensive care setting.

**Data Extraction and Synthesis:**

Of 252 records screened, 20 studies, published from 2004 to 2016, were included. The human factors framework to assess health technologies was applied to measure study completeness, and the Quality Assessment Instrument was used to assess the quality of the studies. PRISMA guidelines were adapted to conduct the systematic review and meta-analysis.

**Main Outcomes and Measures:**

Study completeness and quality; clinician performance; physical, mental, and temporal demand; effort; frustration; time to decision; and decision accuracy.

**Results:**

Of the 20 included studies, 16 were experimental studies with 410 intensive care clinician participants and 4 were survey-based studies with 1511 respondents. Scores for study completeness ranged from 27 to 43, with a maximum score of 47, and scores for study quality ranged from 46 to 79, with a maximum score of 90. Of 20 studies, DIVTs were evaluated in clinical settings in 2 studies (10%); time to decision was measured in 14 studies (70%); and decision accuracy was measured in 11 studies (55%). Measures of cognitive workload pooled in the meta-analysis suggested that any DIVT was an improvement over paper-based data in terms of self-reported performance, mental and temporal demand, and effort. With a maximum score of 22, median (IQR) mental demand scores for electronic display were 10 (7-13), tabular display scores were 8 (6.0-11.5), and novel visualization scores were 8 (6-12), compared with 17 (14-19) for paper. The median (IQR) temporal demand scores were also lower for all electronic visualizations compared with paper, with scores of 8 (6-11) for electronic display, 7 (6-11) for tabular and bar displays, 7 (5-11) for novel visualizations, and 16 (14.3-19.0) for paper. The median (IQR) performance scores improved for all electronic visualizations compared with paper (lower score indicates better self-reported performance), with scores of 6 (3-11) for electronic displays, 6 (4-11) for tabular and bar displays, 6 (4-11) for novel visualizations, and 14 (11-16) for paper. Frustration and physical demand domains of cognitive workload did not change, and differences between electronic displays were not significant.

**Conclusions and Relevance:**

This review suggests that DIVTs are associated with increased integration and consistency of data. Much work remains to identify which visualizations effectively reduce cognitive workload to enhance decision making based on intensive care data. Standardizing human factors testing by developing a repository of open access benchmarked test protocols, using a set of outcome measures, scenarios, and data sets, may accelerate the design and selection of the most appropriate DIVT.

## Key Points

**Question:**

Do data integration and visualization technologies alleviate clinicians’ cognitive workload and alter decision-making performance?

**Findings:**

In this systematic review and meta-analysis of 20 studies, data integration and visualization technologies were associated with improvements in self-reported performance, mental and temporal demand, and effort compared with paper-based recording systems, but no specific type is superior to others. Only 10% of studies of data integration and visualization technology evaluated them in clinical settings.

**Meaning:**

Data integration and visualization technologies offer promising features to improve decision making by clinicians in the intensive care setting, but standardized test protocols are needed to generate clinician-centered evaluations and accelerate screening of technologies that support data-driven decision making.

## Teaser

This systematic review and meta-analysis summarizes the published evidence on the association of user-centered data integration and visualization technologies with intensive care clinician performance.

## Introduction

Advanced monitoring and therapeutic technologies have contributed to improving outcomes in critically ill patients. To make sense of this growing amount of data, technologies tasked with integrating multiple sources of data (ie, devices) must achieve high device interconnectivity and large data storage capacity. Importantly, they must meaningfully display the data. In this regard, technologies need to be both comprehensive and customizable. Data and information visualization is a concept used by different engineering and biological fields faced with large and diverse sources of data.^1^ *Data integration and visualization technology* (DIVT) is a term we use to describe software applications or platforms that integrate continuous data from multiple medical devices. By streaming data from multiple parameters, DIVTs also aim to reduce their multiparametric dimensionality by using algorithms to condense data into new, single-number indicators or visual metaphors that help convey meaning. Efforts to develop technologies that meet these challenging requirements have been underway for decades but have not seen immediate uptake.^2^

The Institute of Medicine’s *To Err is Human: Building a Safer Health System*^3^ suggested that medical error may stem from suboptimal interactions between humans and technology, in part owing to poor technology design. A user-centered design process can ensure that DIVTs safely, effectively, and efficiently support intensive care clinician work.^4^ The challenges of designing these technologies for the delivery of intensive care lie in the highly specialized individual clinicians and the collaborative nature of multidisciplinary team care.^5,6^ Integration not only includes data streams from multiple devices but also the compression of data over the entire length of stay on a single, interactive screen. Data integration and visualization technology is a separate and additional concept to the 24 key functions of comprehensive electronic medical record systems built to serve as repositories.^7^ In 1992, Cunningham et al^8^ introduced MARY, an interactive computerized trend monitoring system, for neonatal intensive care. The display extended seconds-long waveform data of multiple physiological data trends to several minutes or days. Clinicians expected that viewing trends would help manage neonatal care and improve their understanding of patient physiology.^8^ However, in 1991, a randomized clinical trial with MARY as the technological intervention^9^ found no improvement in patient outcomes. An explanation for the system’s ineffectiveness was the “poor presentation of intensive care data [leading] to late or poor interpretation of developing pathology.”^9^ The proposed solution was to make data trends both visually appealing and flexible, ie, to provide a customizable and responsive interface with new visual representations.^9^ A study on visual metaphors of respiratory data^10^ showed that respiratory therapists made decisions twice as fast and with the same level of accuracy compared with traditional displays. These 2 studies did not focus on clinician performance nor did they integrate data from multiple devices. The evolution of formal human factors–centered research in health care and the rapid development of multidevice data integration offer the possibility to evaluate new displays before their clinical deployment. This systematic review aims to determine the association of DIVTs with cognitive performance, particularly clinician decision making, by assessing and synthesizing human factors studies.

## Methods

### Study Identification and Selection

This review’s protocol was conducted by applying the Preferred Reporting Items for Systematic Reviews and Meta-analyses (PRISMA) reporting guideline,^11^ a standard framework in health care research. To our knowledge, there is no PRISMA guided review of human factors research on technology-mediated data integration for the delivery of intensive care.^12,13,14,15^ The study was also registered in PROSPERO (PROSPERO identifier: CRD42015020324).

The systematic search was carried out by a qualified information specialist (C.N.) trained in medical research. Published studies had to fulfill 3 requirements: (1) the study tested a viable DIVT, (2) participants involved were intensive care clinicians, and (3) the study reported quantitative results associated with intensive care decision making. De Georgia et al^2^ indicated computers were broadly introduced to intensive care units (ICUs) in 2003, so our search started in 2004. Searches were conducted in 5 databases (MEDLINE, Embase, Cochrane Central Register of Controlled Trials, PsycINFO, and Web of Science) in May 2014 and were updated in January 2018. Numerous database-specific subject headings were selected to capture the concepts of intensive care, data display, and human factors. The Boolean OR was used to combine all intensive care terms, all data display terms, and all human factor terms. These 3 sets of terms were combined using AND, limited by publication date and to English or French articles. In all databases, truncation and adjacency operators were used to capture word stems and spelling variations. Database subject headings were exploded, when applicable, to include narrower terms. Database used-for terms generated text word searches to combine with the selected database subject headings. The 2 search strategies are included in eMethods 1 and eMethods 2 in the Supplement. Google Scholar was used to complement the systematic search using the terms *human factors*, *data integration*, and *intensive care*. Existing reviews on human factors studies of displays, physiological monitors, and data representations were also screened for additional studies.

### Inclusion and Exclusion Criteria

**Figure 1.**
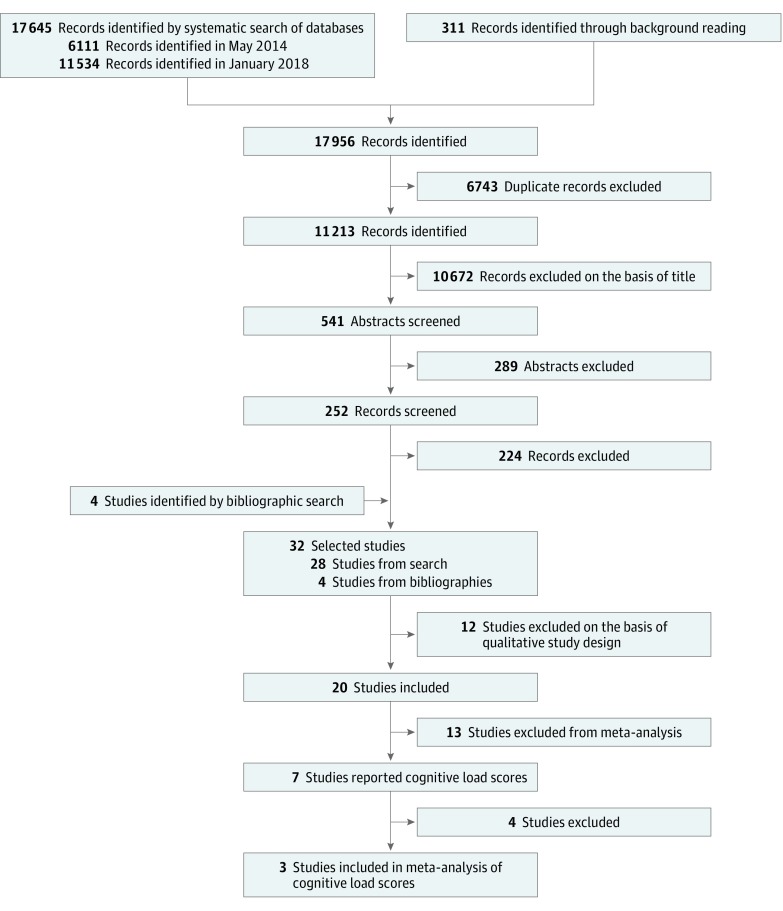
Flow Diagram of Searched, Selected, Included, and Excluded Studies

**Figure 2.**
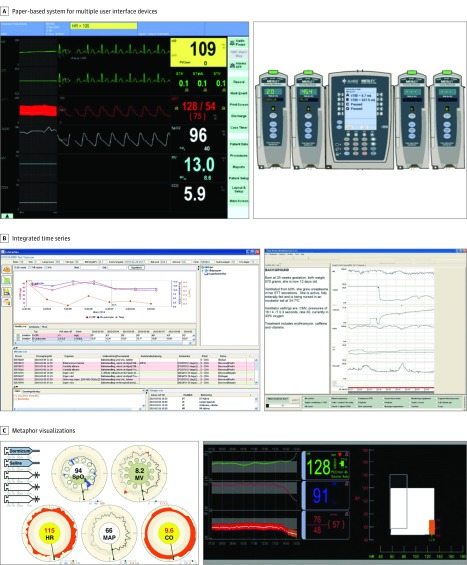
Data Integration and Visualization Technologies A, Paper-based system for multiple-user interface devices. The traditional medical record format comprises mixed records, multiple device interfaces, and free-form text. This system does not integrate patient data from the various sources. Reprinted with permission from Springer Nature.^16^ B, This system, typically an electronic medical record system, provides basic time series of physiological patient data. This system may help understand patient trends over time but may be unsuitable for large numbers of parameters. Reprinted with permission from Taylor & Francis Ltd^17^ and John Wiley and Sons.^18^ C, Physiological data are presented using a metaphor that connects the data being viewed to a real-life device setup (eg, a running infusion) or a physiological system (eg, cardiovascular system). This system relies on the ability of the metaphor to convey meaning and may be a software system separate from an electronic medical record system. Reprinted with permission from Springer Nature^16^ and SAGE Publications, Inc.^19^

Figure 1 illustrates the search and exclusion process. We used inclusion and exclusion criteria to identify veritable human factors studies, searching among randomized clinical trials and observational studies. Studies needed to be original research; to be set in, to simulate, or to have participants from the ICU; and to describe the technology type and its functional capabilities as it related to clinician work. We excluded non-ICU applications, settings, or participants; studies without a prototype DIVT, a focus on explicit sources of data and information, or multiple types of data parameters; and conference articles, editorials, opinion pieces, and reviews. Furthermore, studies must have sought to develop or improve the design of a visualization that integrated continuous and/or intermittent clinical data from explicitly defined sources. Examples of included studies’ DIVTs are shown in Figure 2.^16,17,18,19^ Examples of excluded studies are those that focused on the development of technology without clinician participants (eg, no human factors)^20,21^ or on the technological effect on patient outcomes (eg, length of stay, rates of infection).^22^ For real setting relevance, studies needed a tangible, fully interactive technology with described interface features that explained the association with improved clinician performance. Engineering studies focused on the back-end design of the technology imperceptible to the clinician were excluded. Examples of excluded studies are those that focused on computational advances in data integration and visualization (eg, algorithm development or validation)^23^ or involving medical device interconnectivity.^24,25^

### Review Process

Articles were screened by title, abstract, and full text. Full-text articles were screened independently by 2 authors (Y.L.L. and L.K.) who then selected the final sample. If there was disagreement, articles were discussed for inclusion among 4 authors (Y.L.L., P.T., L.K., and A.-M.G.). The reference lists of these final articles were individually screened for additional articles. The reference management software EndNote (Clarivate Analytics) was used to manage citations.

### Data Extraction and Analysis

The included studies were published from 2004 to 2016. The review and meta-analysis were conducted from May 2014 to April 2018. Two authors (Y.L.L. and L.K.) extracted and tabulated the data from all studies. General study characteristics and appraisal are available in eTable 1 in the Supplement; simulation characteristics and technology characteristics and comparators are found in eTable 2 in the Supplement. Study outcomes based on clinician performance were categorized as positive (statistical improvement), neutral (no statistical difference), or negative (statistical deterioration) (Table).^16,17,18,19,26,27,28,29,30,31,32,33,34,35,36,37,38,39,40,41^ Study completeness and quality were assessed using a modified version of the checklist of essential study elements for human factors studies of health information technologies by Peute et al.^42^ The checklist was modified by adding 2 criteria: (1) if the study received ethics approval and (2) if a Delphi method or another expert consensus process was used. The modified checklist consisted of 47 items and resulted in a maximum score of 47 (eTable 3 in the Supplement). Examples of study requirements include referencing results from previous human factors studies in the Introduction section, including a screenshot of the technology, and providing participants’ level of information technology experience. Discrepancies between abstractors were discussed; the final completeness score was reached through consensus.

**Table.** Metrics and Outcomes Used to Evaluate the Impact of Data Integration and Visualization Technologies on Clinician Performance^a^

Study Name	Task Completion or Decision Time	Task Completion Rate	Time and Accuracy	NASA Task Load Index	Accuracy	Quantity of Information on Screen	Self-reported Usability, Satisfaction, or Preference	No. of Usability Issues	Looking or Accessing Display	System Usability Scale Questionnaire
Ahmed et al,^26^ 2011	Positive	NA	NA	Positive	Positive	Positive	NA	NA	NA	NA
Anders et al,^27^ 2012	NA	NA	NA	Not significant	Positive	NA	Positive	NA	NA	NA
Drews and Doig,^19^ 2014	Positive	NA	NA	Not significant	Positive	NA	Positive	NA	Negative	NA
Dziadzko et al,^28^ 2016	NA	NA	NA	NA	NA	NA	Comparison	NA	NA	NA
Effken,^29^ 2006	Not significant	NA	Not significant	NA	NA	NA	NA	NA	NA	NA
Effken et al,^30^ 2008	Not significant	NA	Not significant	Not significant	NA	NA	Positive	NA	NA	NA
Ellsworth et al,^31^ 2014	NA	NA	NA	NA	NA	NA	Comparison	NA	NA	NA
Forsman et al,^18^ 2013	Comparison	Comparison	NA	NA	NA	NA	Comparison	NA	Comparison	Positive
Görges et al,^32^ 2011	Positive	NA	NA	Not significant	Positive	NA	Positive	NA	NA	NA
Görges et al,^16^ 2012	Positive	NA	NA	Positive	Positive	NA	Positive	NA	NA	NA
Koch et al,^33^ 2013	Positive	NA	NA	NA	Positive	NA	NA	NA	NA	NA
Law et al,^34^ 2005	Not significant	NA	NA	NA	Negative	NA	Positive	NA	NA	NA
Liu and Osvalder,^35^ 2004	Negative	NA	NA	NA	Negative	NA	Positive	NA	NA	NA
Miller et al,^36^ 2009	NA	NA	NA	NA	Positive	NA	NA	NA	NA	NA
Peute et al,^37^ 2011	Positive	Positive	NA	NA	NA	NA	NA	Comparison	NA	NA
Pickering et al,^38^ 2010	Positive	NA	NA	Positive	Positive	NA	NA	NA	NA	NA
Pickering et al,^39^ 2013	NA	NA	NA	NA	NA	Comparison	Comparison	NA	NA	NA
Pickering et al,^40^ 2015	Positive	NA	NA	NA	NA	NA	Positive	NA	NA	NA
van der Meulen et al,^17^ 2010	Not significant	Not significant	NA	NA	Negative	NA	NA	NA	NA	NA
Wachter et al,^41^ 2005	NA	NA	NA	NA	NA	NA	Negative	NA	Positive	NA

Abbreviations: NA, not applicable; NASA-TLX, National Aeronautics and Space Administration Task Load Index.

^a^ Each cell indicates the direction of the result of the tested data integration and visualization technology with a comparator. Positive indicates that the data integration and visualization technology had significantly positive results compared with comparator. Negative indicates that the data integration and visualization technology had negative results compared with a comparator. Not significant indicates that the data integration and visualization technology had no significant results compared with comparator. Comparison indicates that no definitive direction was detected in the results, but that the results associated with each clinician group studied are itemized.

Reporting quality was assessed using a modified version of the Quality Assessment Informatics Instrument (QUASII).^43^ To apply this tool to human factors studies, 3 of the 18 questions were modified: (1) the phrase “implementation of the information system” in item 3 was changed to “technology implementation,” (2) the term “patients” in item 7 was changed to “clinicians,” and (3) the phrase “type of providers” in item 8 was changed to “technology implementation” (eTable 4 in the Supplement). Discriminating between points on the original 7-point scale was challenging owing to the scale as well as to the inappropriateness of the anchor statements to the human factors domain. With a view to diminish perceived subjectivity, the scale was reduced to 5 points. Guided by threats to validity, described by Shadish,^44^ anchor statements for each item were added at the midpoint and 2 end points. Modifications to QUASII were finalized through author consensus prior to the assessment of articles. The maximum modified QUASII score was 90.

### Metrics Used to Evaluate Clinician Performance

Different types of metrics are used in human factors health technology studies to measure the association of the DIVTs with clinician performance. We abstracted the methods used in each study. Metrics of time and decision accuracy include the duration of time to complete a task, time to make a decision, the accuracy of decisions (ie, the rate at which appropriate actions are taken or the rate at which errors are detected), and composite time efficiency and accuracy scores (ie, time within target range). The National Aeronautics and Space Administration Task Load Index (NASA-TLX) is a standardized and validated self-reported measure of cognitive workload consisting of 6 subscales: mental demand, physical demand, temporal demand, performance, effort, and frustration.^45,46^ Usability can be assessed using several methods, including counting and categorizing usability issues; standardized questionnaires including the standard System Usability Scale questionnaire; ad hoc self-reported Likert-type usability scales; or through interviews. Other metrics to measure clinician-technology interaction include measuring the quantity of data elements on the DIVT screen and how often clinicians access the technology.

### Meta-analysis of Standardized Testing Scores

Given the prevalent use of the NASA-TLX, we planned to conduct an individual participant score meta-analysis with NASA-TLX scores that could be retrieved from similar studies. We contacted study authors who used the NASA-TLX to obtain individual participant scores. We used the NASA-TLX scores of a study published on intensive care nursing^47^ as baseline scores to compare paper-based and computerized processes. Each dimension of cognitive load was analyzed separately. The NASA-TLX scores were categorized according to the type of DIVT evaluated: (1) paper control, (2) electronic control, (3) tabular/spreadsheet, and (4) novel visualization. Statistical comparisons of cognitive workload between pairs of displays used Kruskal-Wallis nonparametric testing, using R version 3.4.4 (The R Foundation) with ggplot2, psych, pgirmess, pastecs, and car packages. Tests were 2-tailed, and *P* values less than .05 were considered statistically significant.

## Results

The searches returned a total of 17 645 citations, of which 20 articles satisfied inclusion criteria and were included in this review (Figure 1). The 20 studies consisted of 16 experimental studies^16,17,18,19,26,27,29,30,32,33,34,35,36,37,38,41^ that included 410 intensive care clinician participants and 4 survey-based studies^28,31,39,40^ that included 1511 respondents.

### Completeness and Quality of Studies

**Figure 3.**
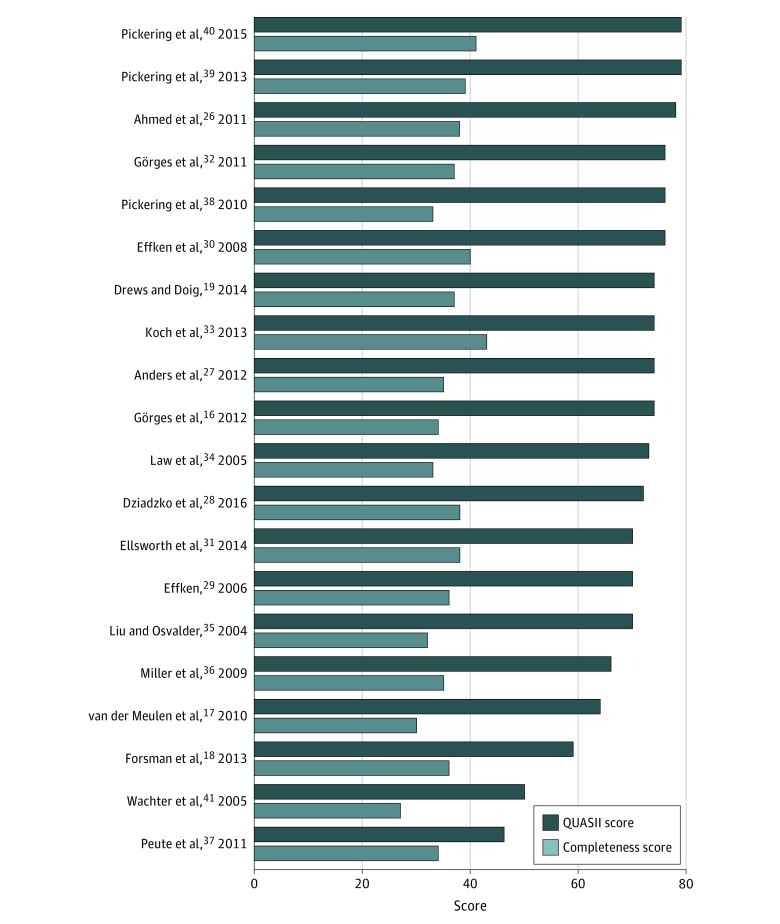
Completeness and Quality Assessment Informatics Instrument (QUASII) Scores Maximum score for completeness is 47; for QUASII, 90.

Study completeness scores ranged from 27 to 43, with a maximum score of 47 (Figure 3). Study quality scores ranged from 46 to 79, with a maximum score of 90 (Figure 3). The lowest QUASII scores were associated with a low degree of technology implementation; other low scores were associated with studies with limited generalizability (eg, to a single setting or participant population) or with minimal consideration for confounders.

### Study Design, Setting, and Participant Characteristics

There were several types of study design, methods, and participant professions. Most studies used a prospective, repeated-measures design. Overall, 14 studies used simulated clinical scenarios, 2 used direct observations,^40,41^ and 1 used ICU live patient data feeds.^38^ Of the 6 studies with scenario descriptions,^19,26,27,29,30,36^ 5 described at least 1 scenario involving sepsis or septic shock.^19,27,29,30,36^

Geographically, 14 studies were conducted in the United States, of which 6 were at the University of Utah and 6 at the Mayo Clinic. Of the remaining studies, 3 were in continental Europe, 2 in the United Kingdom, and 1 in Australia. Overall, 4 studies were conducted at off-site laboratories,^19,30,35,38^ 11 in clinical spaces that used ad hoc simulation rooms,^16,17,18,26,27,29,32,33,34,36,37^ and 2 at the bedside or in the unit.^40,41^ Multiple sites were used in 6 studies.^18,27,28,36,39,40^ In a single study,^18^ 1 site was the prototype development site and the other was the test site.

Half of the studies focused on a single profession (nurses^19,27,30,32,33,35^ or physicians^16,18,26,38^); 4 studies involved both nurses and physicians,^17,29,34,36^ 1 with the addition of respiratory therapists^41^ and 1 with the complete ICU bedside team.^40^ Participant sample size ranged from 6 to 375. When specified, 4 studies specialized in adult patientss,^26,30,36,38^ 2 in neonatal patients,^17,34^ 2 in burn trauma,^18,33^ and 1 in a mix of medical, surgical, and trauma ICUs.^40^ Detailed participant characteristics are reported in eTable 1 in the Supplement.

### Study Technology Characteristics

Data integration and visualization technologies in this review integrated basic vital signs with selected data from infusion pumps,^16,32^ antibiotic use records,^18^ or mechanical ventilation.^35,41^ None of the technologies described in the studies integrated all types of clinical data parameters. Only half were fully functional working prototypes, mimicking actual use and displaying real-time continuous data. From 2004 to 2014, prototype maturity evolved from paper prototypes to fully implemented ICU technologies (Figure 2). For example, in the 2012 study by Görges et al, physicians requested that “systolic and diastolic values be added on the [metaphor visualizations of] mean arterial blood pressure plot of both bar and clock displays.”^16^ Temporal data representation was inferred when study authors used the terms *trending*,^19^ *trajectory*,^27^ or *projection*.^33^

### Study Technology Visualization

Of the 20 studies, 9 compared traditional data displays with novel means of visualization. Computerized natural-language summaries (eg, mimicking physician patient summaries) did not improve decision making compared with time series visualizations.^17,34^ Time series was the primary visual representation of data trends, except for 4 studies that integrated data for patient status assessment.^26,35,38,40^ Metaphor visualizations, which are symbolic representations of parameters or groups of parameters that aim to condense data and are typically non–time series visualizations, were used in 6 studies.^16,19,27,32,35,41^ One study^35^ found that novel metaphor visualizations of physiological and ventilator parameters did not improve problem detection time and created unintended data abstraction that prevented access to higher granularity data.

### Metrics and Outcomes

Among the 20 studies, the 3 most common metrics applied to measure the association with clinician performance were (1) time required to complete a task or make a decision, (2) quality of the decision, and (3) cognitive workload. Time was measured in the context of action (eg, time to initiate decision), waiting (eg, time within target range), or gathering information (eg, time to complete data gathering tasks). Time, as a measure of efficiency, was used in 14 studies (eTable 2 in the Supplement). In 8 of those studies,^16,19,26,32,33,37,38,40^ clinicians were more time efficient with the DIVTs compared with a traditional or previous version of the data information system. In studies by Effken^29^ and Effken et al,^30^ a composite measure of time efficiency and decision accuracy was used but did not show improvement with DIVTs. In another study,^33^ time efficiency and decision accuracy improved at all levels of situation awareness (eg, perception, comprehension, and projection). Measured time, normalized to the system designer’s fastest time to complete a given task, was used in 1 study to compare the novel visualization with its comparator.^37^

The quality of decisions was typically evaluated according to a scorecard developed by experts familiar with the scenarios. Decision-making accuracy was measured in 11 studies.^16,18,26,27,30,32,33,36,37,38,41^ Novel visualizations improved decision accuracy in 8 of 11 studies compared with traditional data information system^16,19,26,27,32,33,38^ or when electronic medical records were compared with paper medical records.^36^ In 3 studies, the subjectivity of scoring decision quality was minimized through independent expert consultation using the Delphi process.^27,30,39^

Report of user preferences (eg, between comparator and DIVTs or open-ended interviews) was used in more than half the studies that provided qualitative data. Overall, 10 studies reported usability or preference on a scale.^16,18,19,27,30,32,34,35,40,41^

### Meta-analysis of the Association of DIVTs With Cognitive Workload

**Figure 4.**
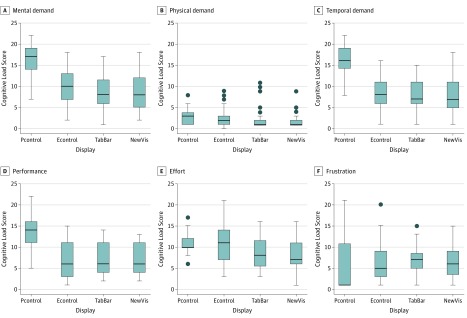
Cognitive Load Across 4 Display Conditions Participant scores of paper control (Pcontrol, 1 study, 26 participants)^47^ were compared with pooled scores of 3 other display conditions: electronic controls (Econtrol, 3 studies, 89 participants),^16,27,32,47^ tabular or bar graphs (TabBar, 3 studies, 63 participants),^16,27,32^ and novel visualizations (NewVis, 3 studies, 63 participants).^16,27,32^ The horizontal line in the box represents the median; the top and bottom borders of the box represent the upper and lower quartiles, respectively; the error bars represent the maximum and minimum values; and the circles outside the box represent outliers (beyond 1.5-fold the interquartile range above the upper quartile or below the lower quartile).

In total, 7 studies used the NASA-TLX to measure self-reported cognitive workload.^16,19,26,27,30,32,38^ The individual participant NASA-TLX scores for 3 studies,^16,27,32^ provided by the investigators of the primary studies, were used to conduct a pooled analysis with an intensive care study measuring cognitive workload using a paper-based system.^47^ The 4 technology categories are the following: (1) paper-based, 1 study, 26 participants^47^; (2) electronic controls, 4 studies, 89 participants^16,27,32,47^; (3) tabular, 3 studies, 63 participants^16,27,32^; and (4) novel visualizations, 3 studies, 63 participants.^16,27,32^ For each category and NASA-TLX dimension, medians and interquartile ranges (IQRs) are shown in Figure 4. Compared with paper-based processes, electronic displays significantly improved mental demand, temporal demand, and performance, suggesting any electronic component or integration of data is superior to a paper system. The median mental demand scores were lower for all electronic visualizations compared with a paper system. With a maximum score of 22, median (IQR) mental demand scores for electronic display were 10 (7-13), tabular display scores were 8 (6.0-11.5), and novel visualization scores were 8 (6-12), compared with 17 (14-19) for paper. The median (IQR) temporal demand scores were also lower for all electronic visualizations compared with paper, with scores of 8 (6-11) for electronic display, 7 (6-11) for tabular and bar displays, 7 (5-11) for novel visualizations, and 16 (14.3-19.0) for paper. The median (IQR) performance scores improved for all electronic visualizations compared with paper (lower score indicates better self-reported performance), with scores of 6 (3-11) for electronic displays, 6 (4-11) for tabular and bar displays, 6 (4-11) for novel visualizations, and 14 (11-16) for paper.

Compared with electronic displays, only self-reported effort significantly improved with tabular and novel visualizations compared with electronic controls, suggesting there is little difference between the electronic visualizations tested. The median (IQR) effort scores were lower for 2 of the electronic displays, with 8 (5.5-11.5) for tabular and bar displays, and 7 (6-11) for novel visualizations, compared with 10 (10-12) for paper. The reported frustration scores were not different for either paper-based or electronic systems.

Of the 4 studies for which we did not obtain individual participant NASA-TLX scores, 2 reported changes in domains^19,30^ and 2 reported changes in the composite score, which combines all 6 domains.^26,38^ Comparing studies reporting on subscales, Effken et al^30^ found an association of DIVTs with performance scores only and none on the other 5 subscales, with a main effect of computer skills. Similarly, Drews and Doig^19^ found an association of DIVTs with mental demand only and none on the other 5 domains.

## Discussion

This human factors systematic review of the association of DIVTs with intensive care clinician performance found published literature using a variety of study designs, methods, task scenarios, and outcome measures. The most common study design was experimental simulation, and the 3 most common assessment metrics were associated with time efficiency, decision accuracy, and cognitive workload. The most mature DIVT was developed through systematic testing,^26,38^ refinement,^28,31^ full-scale implementation, and in 2015, in situ randomized clinical trial evaluation^40^ across a vast ICU network. Human factors testing of DIVTs for intensive care is in its infancy. By design, DIVTs digitize and integrate data and, consequently, organize and format different types of data, ideally into meaningful actionable information. Our findings suggest that the current generation of DIVTs alleviate some facets of cognitive workload on intensive care clinicians simply by unifying data onto a single platform. We did not identify studies of DIVTs that fully integrated all intensive care data streams or addressed the issue of dimensionality reduction (eg, reducing parameters) except through empirical observations to obtain a set of principle variables.^31,42^ While some studies tested visual metaphors, much work still needs to be done to understand which visualization best renders patients’ evolving clinical status.

Our review suggests that published reporting of human factors studies with a high degree of completeness and quality are rare. As new technologies are deployed, rigorous research on their association with decision making would certainly benefit from the use and open access of standard protocols. Sharing methods, including scenario descriptions, test data sets, and scoring metrics, as well as benchmark testing protocols would help developers test DIVTs and new metaphor visualizations. We were able to pool cognitive workload NASA-TLX scores, but this approach could be used with other measures, such as the System Usability Scale questionnaire. The lack of standard scoring metrics highlights the need to standardize reporting methods for the evaluation of clinician-technology interactions and its repercussions on the quality of decision making. By expanding existing data repositories, such as Improving Control of Patient Status in Critical Care (IMPROVE)^16,32,48^ and Multiparameter Intelligent Monitoring in Intensive Care (MIMIC) II,^49^ cohorts of clinical data can target testing DIVTs for different intensive care specialties (eg, cardiac) or professions (eg, respiratory therapists).^50^

This review revealed research gaps in the human factors field for intensive care decision support. This highly specialized health care workforce would benefit from the study of all potential users (eg, respiratory therapists, pharmacists, and dieticians) regarding decision making. Health care professionals beyond nurses and physicians who contribute to the decision-making process and use data from intervention technologies (eg, ventilators, infusion pumps, and parenteral nutrition) need to be studied. Data integration and visualization technologies are emerging from within commercial or comprehensive electronic health records systems, which occasionally offer basic time series data visualizations. These convergent systems should be evaluated, given their growing influence on clinicians’ decision making, using metrics described in this review (eg, time efficiency, accuracy of decision, and cognitive workload).

### Limitations

This study has limitations. We applied a detailed protocol to systematically deconstruct the studies and complete their rigorous appraisal, given their intrinsic limited reporting frameworks. The PRISMA item related to bias rating was not done. The 12-year publishing period of 2004 to 2016 may introduce heterogeneity (and antiquated comparators). However, we speculate that the slow cycles of implementation of health care technologies may hold our conclusions still relevant for most hospitals today. The key limitation of most of the studies was a control group that did not comprehensively represent the complete clinical information system (eg, paper medical records, electronic medical records system, and other dedicated monitors). In reality, the information system may be combinations of an electronic health record, paper medical records, other stand-alone monitoring and intervention devices, and so on. Identifying these technological components and designing human factors studies that compare all sources with those integrated in the novel DIVT would support divestment from unnecessary technologies. Studies with positive results are disproportionally represented in this review compared with negative or not significant results, which suggests a publication bias.

## Conclusions

This review summarizes the measured association of DIVTs with intensive care clinician performance. Cognitive workload, measured using the NASA-TLX instrument, was the only measure that indicated that any form of electronic data integration and visualization was associated with an improvement in the process compared with paper-based systems. This review also presents a variety of technologies, study designs, clinician participants, settings, scenarios, tasks, comparators, and outcome measures. We propose that sharing scenarios, test data sets, and customized metrics could greatly speed up the process of human factors testing of novel DIVTs for different ICU specialties. Our findings suggest that best practice human factors evaluation metrics should be used to measure changes in decision-making efficiency and to harmonize data to make them amenable to aggregation using meta-analysis techniques.

## Notes

Supplement. **eMethods 1.** MEDLINE Example: Search Strategy Conducted in May 2014 **eMethods 2.** Web of Science Example: Search Strategy Conducted in January 2018 **eTable 1.** Characteristics of the Studies, Technology, Study Completeness Score, and QUASII Score **eTable 2.** Study Design Type, Scenario Descriptions, Tasks, Technology Maturity, Comparators, and Temporal Representations **eTable 3.** Modified Study Completeness Assessment Tool for Quantitative Human Factors Studies **eTable 4.** Human Factors Study Quality Assessment Tool, a Modified Version of the Quality Assessment Informatics Instrument (QUASII) **eReferences**
