# Supplementary material for: Association of Data Integration Technologies With Intensive Care Clinician Performance: A Systematic Review and Meta-analysis
Source: JAMA Netw Open. 2019 May 24;2(5):e194392. doi: 10.1001/jamanetworkopen.2019.4392 (PMC6632132; doi:10.1001/jamanetworkopen.2019.4392)
Supplement: Supplement. — eMethods 1. MEDLINE Example: Search Strategy Conducted in May 2014 eMethods 2. Web of Science Example: Search Strategy Conducted in January 2018 eTable 1. Characteristics of the Studies, Technology, Study Completeness Score, and QUASII Score eTable 2. Study Design Type, Scenario Descriptions, Tasks, Technology Maturity, Comparators, and Temporal Representations eTable 3. Modified Study Completeness Assessment Tool for Quantitative Human Factors Studies eTable 4. Human Factors Study Quality Assessment Tool, a Modified Version of the Quality Assessment Informatics Instrument (QUASII) eReferences [file jamanetwopen-2-e194392-s001.pdf]

Supplementary Online Content

Lin YL, Trbovich P, Kolodzey L, Nickel C, Guerguerian A-M. Association of data integration technologies with intensive care clinician performance: a systematic review and meta-analysis. *JAMA Netw Open.* 2019;2(5):e194392. doi:10.1001/jamanetworkopen.2019.4392

eMethods 1. MEDLINE Example: Search Strategy Conducted in May 2014
eMethods 2. Web of Science Example: Search Strategy Conducted in January 2018
eTable 1. Characteristics of the Studies, Technology, Study Completeness Score, and QUASII Score
eTable 2. Study Design Type, Scenario Descriptions, Tasks, Technology Maturity, Comparators, and Temporal Representations
eTable 3. Modified Study Completeness Assessment Tool for Quantitative Human Factors Studies
eTable 4. Human Factors Study Quality Assessment Tool, a Modified Version of the Quality Assessment Informatics Instrument (QUASII)
eReferences

This supplementary material has been provided by the authors to give readers additional information about their work.

© 2019 Lin YL et al. *JAMA Network Open.*

eMethods 1. Medline Example: Search Strategy Conducted in May 2014

Databases: Medline, Embase, Web of Science, PsycINFO

1 exp Critical Care/ (43531)
2 exp Intensive Care Units/ (55611)
3 ((critical or intensive) adj2 care).mp. (130846)
4 (PICU or NICU or ICU).mp. (32491)
5 ((patient* or ambulator* or "body temperature*" or electrocardiograph* or ekg or ecg or "electric cardiogram*" or electrocardiogram* or brain* or cerebral*) adj2 monitor*).mp. (39649)
6 ("life support*" or CPR or resuscitat* or reanimat* or capnogra* or capnometry or neuromonitor* or telemonitor*).mp. (72347)
7 ((airway* or breath*) adj2 (control* or manage* or regulat*)).mp. (9010)
8 (high adj2 frequenc* adj2 ventilat*).mp. (3582)
9 ((invasive or noninvasive or "non invasive") adj2 ventilat*).mp. (3469)
10 (pressure* adj2 (breath* or respirat* or ventilat*)).mp. (23265)
11 (respirat* adj2 (control* or regulat*)).mp. (6807)
12 (therapeutic* adj2 hyperventilat*).mp. (10)
13 (ventilat* adj2 support).mp. (5872)

14 or/1-13 (280140)

15 (data* adj2 display*).mp. (7541)
16 (physiologic* adj2 monitor*).mp. (45714)
17 (graph* adj2 display*).mp. (1509)
18 (data* adj2 interface*).mp. (586)
19 (monitor* adj2 (system* or platform*)).mp. (12029)
20 (software* adj2 (system* or platform*)).mp. (2698)
21 ((multimodal* or multi-modal*) adj2 monitor*).mp. (259)
22 (continuous adj2 monitor*).mp. (9941)
23 (computer* adj2 (design* or graphic*)).mp. (25269)
24 (software* adj2 design*).mp. (6419)
25 informatic*.mp. (17315)
26 (data* adj2 acquisition*).mp. (8040)
27 (integrat* adj2 (display* or monitor* or platform* or software*)).mp. (1802)

28 or/15-27 (129019)

29 evaluation studies as topic/ or device approval/ or diagnostic test approval/ or feasibility studies/ or pilot projects/ or program evaluation/ or validation studies as topic/ (284249)
30 decision support techniques/ or data interpretation, statistical/ (60230)
31 Decision Support Systems, Clinical/ (4743)
32 Technology Assessment, Biomedical/ (8069)
33 "outcome assessment (health care)"/ or patient outcome assessment/ or watchful waiting/ or "process assessment (health care)"/ (52518)
34 quality assurance, health care/ or total quality management/ or quality improvement/ (64251)
35 user-computer interface/ (27480)
36 Adaptation, Psychological/ (73905)
37 human engineering/ or man-machine systems/ or "task performance and analysis"/ or "time and motion studies"/ or work simplification/ (36767)
38 Consumer Satisfaction/ (17443)
39 human factor*.mp. (4523)
40 (adapt* or adjust* or analys* or assess* or "clinical prediction*" or coping or "critical incident techni*" or critique* or "decision aid*" or "decision* model*" or "decision* support model*" or "decision* support system*" or "decision* support techni*" or "device approv*" or "diagnostic test* approv*" or effective* or ergonomic* or

© 2019 Lin YL et al. *JAMA Network Open.*

evaluat* or "feasibility stud*" or "human engineer*" or interpret* or "man-machine system*" or outcome* or "pilot project*" or "pilot stud*" or preference* or "pre-post test*" or "process* measure*" or "program* sustainab*" or "psychology engineer*" or "quality assurance" or "quality improve*" or "quality manage*" or satisf* or "task* performance*" or "technology assess*" or "time and motion stud*" or "time stud*" or "user-computer interface*" or "validation stud*" or "virtual system*" or "watchful wait*" or "work simplif*").mp. (7676889)

41 or/29-40 (7678638)

42 14 and 28 and 41 (10389)

43 limit 42 to yr="2000 -Current" (6209)

© 2019 Lin YL et al. *JAMA Network Open.*

eMethods 2. Web of Science Example: Search Strategy Conducted in January 2018

Databases: Medline, Embase, Web of Science, PsycINFO.

Set	Results	
# 38	1,794	(#35 OR #28) AND LANGUAGE: (English)<br>Indexes=SCI-EXPANDED, SSCI, CPCI-S, CPCI-SSH Timespan=2014-2018
# 37	5,283	(#35 OR #28) AND LANGUAGE: (English)<br>Indexes=SCI-EXPANDED, SSCI, CPCI-S, CPCI-SSH Timespan=All years
# 36	5,495	#35 OR #28<br>Indexes=SCI-EXPANDED, SSCI, CPCI-S, CPCI-SSH Timespan=All years
# 35	171	#34 AND #7<br>Indexes=SCI-EXPANDED, SSCI, CPCI-S, CPCI-SSH Timespan=All years
# 34	29,939	#33 OR #32 OR #31 OR #30 OR #29<br>Indexes=SCI-EXPANDED, SSCI, CPCI-S, CPCI-SSH Timespan=All years
# 33	14,704	TS=(human* NEAR/2 computer* NEAR/2 (interface* or interact*))<br>Indexes=SCI-EXPANDED, SSCI, CPCI-S, CPCI-SSH Timespan=All years
# 32	737	TS="man-machine system*"<br>Indexes=SCI-EXPANDED, SSCI, CPCI-S, CPCI-SSH Timespan=All years
# 31	12,259	TS=(graph* NEAR/1 user* NEAR/1 interface*)<br>Indexes=SCI-EXPANDED, SSCI, CPCI-S, CPCI-SSH Timespan=All years
# 30	2,300	TS=(ecological NEAR/2 (display* or interface* or monitor*))<br>Indexes=SCI-EXPANDED, SSCI, CPCI-S, CPCI-SSH Timespan=All years
# 29	333	TS="user-computer interface*"<br>Indexes=SCI-EXPANDED, SSCI, CPCI-S, CPCI-SSH Timespan=All years
# 28	5,395	#27 AND #23 AND #7<br>Indexes=SCI-EXPANDED, SSCI, CPCI-S, CPCI-SSH Timespan=All years
# 27	18,330,350	#26 OR #25 OR #24<br>Indexes=SCI-EXPANDED, SSCI, CPCI-S, CPCI-SSH Timespan=All years
# 26	2,601,342	TS=simulat*<br>Indexes=SCI-EXPANDED, SSCI, CPCI-S, CPCI-SSH Timespan=All years
# 25	16,751,072	TS=(adapt* or adjust* or analys* or assess* or "clinical prediction*" or "critical incident techni*" or critique* or "decision aid*" or "decision* model*" or "decision* support model*" or "decision* support system*" or "decision* support techni*" or effective* or ergonomic* or evaluat* or "human engineer*" or interpret* or outcome* or preference* or "process* measure*" or "psychology engineer*" or satisf* or "task* performance*" or "technology assess*" or "time and motion stud*" or "time stud*" or "watchful wait*" or "work simplif*")<br>Indexes=SCI-EXPANDED, SSCI, CPCI-S, CPCI-SSH Timespan=All years
# 24	662,501	TS=human factor*<br>Indexes=SCI-EXPANDED, SSCI, CPCI-S, CPCI-SSH Timespan=All years
# 23	406,575	#22 OR #21 OR #20 OR #19 OR #18 OR #17 OR #16 OR #15 OR #14 OR #13 OR #12 OR #11 OR #10 OR #9 OR #8<br>Indexes=SCI-EXPANDED, SSCI, CPCI-S, CPCI-SSH Timespan=All years
# 22	83,732	TS=(information NEAR/2 technolog*)<br>Indexes=SCI-EXPANDED, SSCI, CPCI-S, CPCI-SSH Timespan=All years
# 21	15,777	TS=((data* or information*) NEAR/2 visualization)<br>Indexes=SCI-EXPANDED, SSCI, CPCI-S, CPCI-SSH Timespan=All years
# 20	21,211	TS=(integrat* NEAR/2 (display* or monitor* or platform* or software*))<br>Indexes=SCI-EXPANDED, SSCI, CPCI-S, CPCI-SSH Timespan=All years
# 19	54,416	TS=(data* NEAR/2 acquisition*)<br>Indexes=SCI-EXPANDED, SSCI, CPCI-S, CPCI-SSH Timespan=All years
# 18	20,236	TS=informatic*

© 2019 Lin YL et al. *JAMA Network Open.*

		Indexes=SCI-EXPANDED, SSCI, CPCI-S, CPCI-SSH Timespan=All years
# 17	27,330	TS=(software* NEAR/2 design*)<br>Indexes=SCI-EXPANDED, SSCI, CPCI-S, CPCI-SSH Timespan=All years
# 16	43,047	TS=(computer* NEAR/2 (design* or graphic*))<br>Indexes=SCI-EXPANDED, SSCI, CPCI-S, CPCI-SSH Timespan=All years
# 15	25,505	TS=(continuous NEAR/2 monitor*)<br>Indexes=SCI-EXPANDED, SSCI, CPCI-S, CPCI-SSH Timespan=All years
# 14	606	TS=((multimodal* or multi-modal*) NEAR/2 monitor*)<br>Indexes=SCI-EXPANDED, SSCI, CPCI-S, CPCI-SSH Timespan=All years
# 13	48,254	TS=(software* NEAR/2 (system* or platform*))<br>Indexes=SCI-EXPANDED, SSCI, CPCI-S, CPCI-SSH Timespan=All years
# 12	76,107	TS=(monitor* NEAR/2 (system* or platform*))<br>Indexes=SCI-EXPANDED, SSCI, CPCI-S, CPCI-SSH Timespan=All years
# 11	7,252	TS=(data* NEAR/2 interface*)<br>Indexes=SCI-EXPANDED, SSCI, CPCI-S, CPCI-SSH Timespan=All years
# 10	6,151	TS=(graph* NEAR/2 display*)<br>Indexes=SCI-EXPANDED, SSCI, CPCI-S, CPCI-SSH Timespan=All years
# 9	3,764	TS=(physiologic* NEAR/2 monitor*)<br>Indexes=SCI-EXPANDED, SSCI, CPCI-S, CPCI-SSH Timespan=All years
# 8	7,941	TS=(data* NEAR/2 display*)<br>Indexes=SCI-EXPANDED, SSCI, CPCI-S, CPCI-SSH Timespan=All years
# 7	209,300	#6 OR #5 OR #4 OR #3 OR #2 OR #1<br>Indexes=SCI-EXPANDED, SSCI, CPCI-S, CPCI-SSH Timespan=All years
# 6	1,749	TS=(med* NEAR/2 surg* NEAR/2 unit*)<br>Indexes=SCI-EXPANDED, SSCI, CPCI-S, CPCI-SSH Timespan=All years
# 5	16,384	TS=(pressure* NEAR/2 (breath* or respirat* or ventilat*))<br>Indexes=SCI-EXPANDED, SSCI, CPCI-S, CPCI-SSH Timespan=All years
# 4	1,384	TS=neuromonitor*<br>Indexes=SCI-EXPANDED, SSCI, CPCI-S, CPCI-SSH Timespan=All years
# 3	36,275	TS=((patient* or brain* or cerebral*) NEAR/2 monitor*)<br>Indexes=SCI-EXPANDED, SSCI, CPCI-S, CPCI-SSH Timespan=All years
# 2	51,821	TS=(PICU or NICU or ICU)<br>Indexes=SCI-EXPANDED, SSCI, CPCI-S, CPCI-SSH Timespan=All years
# 1	143,145	TS=((critical or intensive) NEAR/2 care)<br>Indexes=SCI-EXPANDED, SSCI, CPCI-S, CPCI-SSH Timespan=All years

© 2019 Lin YL et al. *JAMA Network Open.*

eTable 1. Characteristics of the Studies, Technology, Study Completeness Score and QUASII Score

Study completeness score, max 47; QUASII score, max 90. Studies ordered alphabetically by last name of the first author and year of publication.

Study	Last name (year)	Country/ Setting	Sample	Technology	Study Design/Methods/Measures	Key Findings	Completeness Score	QUASII Score
1	Ahmed (2011)	USA,<br>ICU in an academic tertiary referral center	Six attending physicians, 14 residents/fellows	Novel user interface electronic environment<br>Task: specific user interfaces<br>EMR: user interface showing	Design: Prospective, unblinded randomized crossover study<br>Methods: Low-fidelity simulation<br>Measures: Cognitive workload (NASA-TLX), number of errors in cognition, time to task completion (in seconds), total quantity of data presented	Cognitive workload, error rates, and time efficiency improved while data points were reduced with novel technology	38	78
2	Anders (2012)	USA,<br>two university teaching hospital intensive care units	32 ICU nurses, 16 per site	Integrated graphical information display	Design: Repeated measures simulations study<br>Methods: Low-fidelity simulation<br>Measures: Percent correct detection of abnormal patient variables, nurse task load (NASA-TLX), display usability rating	Clinicians were able to better detect errors with the novel technology	35	74

© 2019 Lin YL et al. *JAMA Network Open.*

Study	Last name (year)	Country/ Setting	Sample	Technology	Study Design/Methods/Measures	Key Findings	Completeness Score	QUASII Score
3	Drews (2014)	USA, applied and basic cognition laboratory	42 ICU nurses	Configural Vitals Signs (CVS) Display	Design: Between-subjects experimental design<br>Method: Low-fidelity simulation<br>Measures: Response time, accuracy of data interpretation, workload (NASA-TLX), clinical desirability of CVS display	Data interpretation and accuracy improved with the novel technology	37	74
4	Dziadzko (2016)	USA, ICUs at two tertiary hospitals	246 respondents before (existing EMR) and 115 respondents after (existing EMR+ novel EMR interface) surveys	AWARE (ambient warning and response evaluation), a novel internet based application extracts and organizes relevant patient data using ranking and decision rules	Design: Before and after implementation survey<br>Methods: Survey<br>Measures: User satisfaction and usability	Clinicians with prescribing duties preferred the novel technology	38	72
5	Effken (2006)	USA, Arizona Health Sciences Center	20 novice ICU nurses, 13 medical residents with ICU rotation, three expert ICU nurses, three expert ICU physicians	Etiotlogic Potentials Display (EPD)	Design: Mixed design two (order) × two (display) × four (scenario) × three (level)<br>Methods: High-fidelity simulation with interactive simulator<br>Measures: Treatment initiation time, percentage of	Clinician performance did not improve with novel interface. Clinicians preferred data displayed as	36	70

© 2019 Lin YL et al. *JAMA Network Open.*

Study	Last name (year)	Country/ Setting	Sample	Technology	Study Design/Methods/Measures	Key Findings	Completeness Score	QUASII Score
					time patient variables were kept within a target range	numerical values		
6	Effken (2008)	USA, University of Arizona	32 adult ICU nurses	Ecological Display (ED) with variables ordered	Design: Mixed experimental design<br>Methods: Written pretests and simulation<br>Measures: Critical event recognition, treatment efficiency, cognitive workload, user satisfaction	If too many pictorial representations are used, efficient clinical decision-making may be impeded	40	76
7	Ellsworth (2014)	USA, neonatal ICU, Mayo Clinic	23 NICU respondents: eight attending physicians, two fellows, four nurse practitioners, nine pediatric residents	98 unique data items available from the EMR	Design: Web-based survey<br>Methods: Web-based survey<br>Measures: Mean importance score for data items used in NICU routine clinical decision making	Top five data items: daily weight, pH, partial pressure of carbon dioxide (pCO_2), fraction of inspired oxygen (FiO_2) and blood culture results.<br>16 of the 98 items (16%) received an absolutely necessary rating by more than 50% of respondents	38	70

© 2019 Lin YL et al. *JAMA Network Open.*

Study	Last name (year)	Country/ Setting	Sample	Technology	Study Design/Methods/Measures	Key Findings	Completeness Score	QUASII Score
8	Forsman (2013)	Sweden, ICUs of a university hospital and two general hospitals	15 physicians (ethnographic study), eight physicians (usability testing)	Integrated information display for patient infection status to inform antibiotics use	Design: Ethnographic studies and participatory design<br>Methods: Observations, semi-structured interviews, focus group, usability testing with eye tracking<br>Measures: Time, System Usability Scale questionnaire	Physicians preferred novel technology compared to existing system, mean SUS was 79.5%	36	59
9	Görges (2011)	USA, University of Utah Health Sciences Center break room	16 medical ICU nurses	Two far-view physiological monitoring displays:<br>- strip-chart/bar display<br>- circular, clock-like display	Design: Repeated-measures within-subject experimental design<br>Method: Low-fidelity simulation, randomized repeated measures within-subject design<br>Measures: Decision time, decision accuracy, workload scores, display preference	Time, accuracy, cognitive workload improved with novel technology	37	76
10	Görges (2012)	USA University of Utah Health Sciences Center, break room	15 ICU fellow physicians	Two far-view physiological monitoring displays:<br>- strip-chart/bar display<br>- circular, clock-like display	Design: Randomized repeated measures within-subject design<br>Method: Low-fidelity simulation<br>Measures: Decision time, decision accuracy, workload scores, display preference	Time, accuracy and cognitive workload improved with novel technology	34	74
11	Koch (2013)	USA, nurses' break room, BTICU	12 experienced BTICU nurses	Integrated information display with information used for	Design: Counter-balanced (on display order), repeated-measures design<br>Method: Simulations requiring participants identify	Situation awareness improved with novel technology	43	74

© 2019 Lin YL et al. *JAMA Network Open.*

Study	Last name (year)	Country/ Setting	Sample	Technology	Study Design/Methods/Measures	Key Findings	Completeness Score	QUASII Score
				comparable tasks was shown in spatial proximity	information about medication management, patient awareness, and team communication<br>Measures: Situation awareness (accuracy of the participants' answer) and task completion time			
12	Law (2005)	UK, Neonatal ICU	32 nurses and 16 physicians	Displays presented in a research version of Badger trend monitoring system	Design: Mixed design<br>Method: Simulations requiring participants perform eight types of actions: order chest X-ray, intubate or re-intubate, re-apply transcutaneous probe, start dopamine, treat with surfactant, put patient on high-frequency oscillatory ventilation, start continuous positive airway pressure, or no action<br>Measures: Speed of responses, quality/appropriateness of responses, reported preference	Clinicians selected more appropriate actions when data presented in text form versus graph but time efficiency was similar	33	73
13	Liu (2004)	Sweden, ICU of university hospital and usability laboratory	Interviews: Six ICU nurses<br>Usability testing: In 20 medical nursing students	Graphical circular display prototype of a ventilator machine display	Design: Within subject design<br>Method: Interviews and usability testing<br>Measures: Detection time and error rates	Detection time and error rates did not improve significantly with novel technology	32	70

© 2019 Lin YL et al. *JAMA Network Open.*

Study	Last name (year)	Country/ Setting	Sample	Technology	Study Design/Methods/Measures	Key Findings	Completeness Score	QUASII Score
14	Miller (2009)	Australia, ICUs of two major metropolitan tertiary teaching hospitals	16 nurses and 12 physicians	Work Domain Analysis-based Paper Prototype and Electronic Prototype of a clinical information system	Design: Within-participants, two (control and prototype) x four (patient data sets) counterbalanced design<br>Methods: Simulated scenarios where nurses detected changes in patient parameters and physicians completed diagnostic tasks<br>Measures: Detection of patient change (nurses) and physician diagnostic agreement	Detection of patient change (nurses) and diagnostic agreement (physicians) improved with novel technology	35	66
15	Peute (2011)	The Netherlands, clinical workspace	12 participants (unspecified specialty)	Web-based data query tool of the Dutch National ICE	Design: Pre-post design, think-aloud usability study<br>Method: Usability testing<br>Measures: Number of usability issues and task efficiency	Task completion and efficiency improved after technology interface redesign	34	46
16	Pickering (2010)	USA, remote testing facility	Six off-duty critical care fellows and residents	AWARE program which extracts a subset of predefined cues based on relevance	Design: Prospective, randomized, crossover pilot study<br>Methods: Simulated scenarios to extract a sequence of decision making cues<br>Measures: Cognitive workload (NASA TLX), number of medical error and efficiency (time and accuracy)	Novel technology reduced time to task completion, provider cognitive workload and medical error	33	76

© 2019 Lin YL et al. *JAMA Network Open.*

Study	Last name (year)	Country/ Setting	Sample	Technology	Study Design/Methods/Measures	Key Findings	Completeness Score	QUASII Score
17	Pickering (2013)	USA, three ICUs	1,277 physician-patient interactions and 925 respondents	Institutional EMR system integrating vital signs, microbiology, medications, laboratory results, fluids, nursing flow sheet items, and clinical notes	Design: Prospective observational study and retrospective chart review<br>Methods: Observations and questionnaires<br>Measures: Frequency of data elements used per physician-patient interaction episode.	Over 25% of the information contained in the EMR was never used for diagnosis and treatment. Monitoring and lab data was most valuable	39	79
18	Pickering (2015)	USA, four ICUs	375 clinicians (physicians, nurses, respiratory therapists and pharmacists), 169 survey respondents	AWARE program deployed in the ICUs compared to EMR system	Design: Step wedge cluster randomized control trial<br>Methods: Direct observations and surveys<br>Measures: Time to gather information	Time spent on pre-round data gathering per patient decreased with novel technology	41	79
19	van der Meulen (2010)	UK, Neonatal ICU	18 physicians and 17 nurses	Natural Language Generation using BT-45 computer program, summarizing physiological data	Design: Mixed experimental design<br>Methods: Simulations where participants must select appropriate actions<br>Measures: Response time and scores through expert consensus whether clinicians' actions were appropriate, inappropriate, and neutral	Human generated descriptions were better able to support medical decision-making than graphs with trend lines. Computer text condition was comparable to graph condition	30	64

© 2019 Lin YL et al. *JAMA Network Open.*

Study	Last name (year)	Country/ Setting	Sample	Technology	Study Design/Methods/Measures	Key Findings	Completeness Score	QUASII Score
20	Wachter (2005)	USA, Medical ICU, University of Utah Hospital	32 clinicians (physicians, residents, nurses, and respiratory therapists) attending to two ventilator-dependent patients	Pulmonary function graphical and numerical display with fraction inspired oxygen (FiO_2), end tidal carbon dioxide, tidal volume, and anatomical representation of intrinsic positive end expiratory pressure	Design: Observational study design<br>Methods: Observations and questionnaires<br>Measures: Number of glances at display, perceived usefulness, acceptance, desirability and accuracy of display	Nurses, respiratory therapists, and physicians looked at the novel technology, on average 1.31, 3 and 6, times per visit	27	50

Abbreviations: AWARE, ambient warning and response evaluation; Badger; BTICU, burn trauma intensive care unit; CVS, configurable vital signs; ED, ecological display; EMR, electronic medical record; EP, electronic prototype; EPD, etiologic potentials display; ICU, intensive care unit; NASA TLX, National Aeronautics and Space Administration Task Load Index; NLG, Natural Language Generation; PP, paper prototype; QUASII, QUality ASessment Informatics Instrument; SUS, system usability scale; USA, United States of America; WDA, work domain analysis.

© 2019 Lin YL et al. *JAMA Network Open.*

eTable 2. Study Design Type, Scenario Descriptions, Tasks, Technology Maturity, Comparators, and Temporal Representations

Each column indicates a study ordered alphabetically by last name of the first author and year of publication. The number ‘1’ in a cell indicates the information was reported in the study’s manuscript. In Total column, we provide the sum of studies with the feature in the corresponding row.

**Study**	**Last name first author (year)**																				
**Number**	**1**	**2**	**3**	**4**	**5**	**6**	**7**	**8**	**9**	**10**	**11**	**12**	**13**	**14**	**15**	**16**	**17**	**18**	**19**	**20**	
	**Ahmed (2011)**	**Anders (2012)**	**Drews (2014)**	**Dziadzko 2016)**	**Effken (2006)**	**Effken (2008)**	**Ellsworth (2014)**	**Forsman (2013)**	**Görges (2011)**	**Görges (2012)**	**Koch (2013)**	**Law (2005)**	**Liu (2004)**	**Miller (2009)**	**Peute (2011)**	**Pickering (2010)**	**Pickering (2013)**	**Pickering (2015)**	**van der Meulen (2010)**	**Wachter (2005)**	**Total**
**Design**																					
Simulation	1	1	1		1	1		1	1	1	1	1	1	1	1						14
In-situ/direct observations																1		1		1	3
Questionnaire/ survey				1			1										1				3
Study assessment of realism			1								1										2
**Scenario descriptions (when reported)**																					
Sepsis/septic shock		1	1		1	1								1							5
Pulmonary embolus/edema			1											1							2
Stable patient			1																		1
Actively bleeding	1																				1
Post-operation					1	1								1							3
Acute resp. distress syndrome					1									1							2
Abnormal cardiac rhythm					1	1															2
Tamponade					1																1

© 2019 Lin YL et al. *JAMA Network Open.*

**Study**	**Last name first author (year)**																				
**Number**	**1**	**2**	**3**	**4**	**5**	**6**	**7**	**8**	**9**	**10**	**11**	**12**	**13**	**14**	**15**	**16**	**17**	**18**	**19**	**20**	
	**Ahmed (2011)**	**Anders (2012)**	**Drews (2014)**	**Dziadzko 2016)**	**Effken (2006)**	**Effken (2008)**	**Ellsworth (2014)**	**Forsman (2013)**	**Görges (2011)**	**Görges (2012)**	**Koch (2013)**	**Law (2005)**	**Liu (2004)**	**Miller (2009)**	**Peute (2011)**	**Pickering (2010)**	**Pickering (2013)**	**Pickering (2015)**	**van der Meulen (2010)**	**Wachter (2005)**	**Total**
**Tasks**																					
Use of antibiotics								1													1
Mechanical ventilation													1							1	2
Database query															1						1
Continuous infusions									1	1	1										3
**Technology maturity**																					
Paper														1							1
Computer static														1						1	2
Computer slide deck with some interaction			1		1				1	1	1	1	1						1		8
Fully-interactive	1	1				1		1							1	1	1	1			8
**Comparator**																					
Traditional data source	1		1						1	1	1		1	1		1		1			9
Other display					1	1															2
Other specialty								1												1	2
**Temporal representation**																					
Current	1				1	1					1		1			1	1	1		1	9
Historical (explicit)								ICU stay	12h	12h		30min, 53min		5 days			3h		45min		7
Historical (implicit)		1	1								1										3

© 2019 Lin YL et al. *JAMA Network Open.*

eTable 3. Modified Study Completeness Assessment Tool for Quantitative Human Factors Studies

The original study completeness checklist published by Peute et al. consisted of 51 items.[1] The practical application of the checklist resulted in the removal of six items (items strike through 13, 30, 33, 43, 44 and 45) and the addition of two items (marked as new in the item # column). The maximum completeness score is 47 with one point for each element.

Heading	Sub-Heading	Item #	Item description
Introduction	Keywords	1	Type or functionality of the system
		2	User-Centered Design phases
		3	Methods applied
		4	Usability as mesh term
	Essential Information	5	Conclusion or recommendations previous Human Factors/usability studies
		6	Purpose and reason for study
		7	Scientific aims
Background information	If Human Factors/usability study is an integrated part in Health Information Technology development	8	User-Centered Design phases that are covered
		9	System design principles or existing standards used specifications/goals/requirements depending on User-Centered Design phases
	If the study is scientifically oriented	10	User interface design principles applied or methods evaluated
		11	Theories underlying the interface design principles or methods evaluated
	System type or its part/functionality	12	Version
		~~13~~	~~Release date (removed)~~
		14	Graphical view
		15	The setting
		16	The user tasks to be supported
		17	Overview actual/intended users' profile
		18	If the system is in use the context of the system
		19	User characteristics
		20	Organizational and physical environment and equipment
Method	Methods section	21	Applied method(s)
		22	Suitability of each method
		23	Number of and expertise background of study evaluators
		24	Description of study variables
		25	Outcome measures and quality metrics
		26	If study used test scenarios or tasks
		new	If scenarios developed based on Delphi or expert consensus
		27	If study participants are (representative) end users

© 2019 Lin YL et al. *JAMA Network Open.*

		new	Institutional Research Board or Research Ethics Board approval
	Background study participants	28	Age
		29	Gender
		~~30~~	~~Linguistic and culture background (removed)~~
		31	Level of education
		32	Professional competence
		~~33~~	~~potential disabilities (removed)~~
		34	Level of experience using Information Technology
		35	Level of experience with similar system
	Generalizability and reproducibility of the study	36	Setting of the study
		37	Study period and evaluation time
		38	Instructions provided to participants and the recruitment
Results	Result section	39	If Human Factors/usability methods have been applied
		40	Results are reported on per method
		41	Unexpected events encountered
		42	Unexpected results uncovered
	If the study reports on usability problems	~~43~~	~~Presentation of results should rely on classification scheme (removed)~~
		~~44~~	~~usability problems rated for their severity (removed)~~
		~~45~~	~~usability problems rated for their potential impact on patient safety (removed)~~
Discussion		46	Intended purpose of the study is achieved
		47	Limitations of the study
		48	Contribution of the study to the User-Centered Design process
		49	Added value of method applied
		50	Knowledge/evidence gained in terms of Human Factors/usability principles
		51	Added value of this paper

© 2019 Lin YL et al. *JAMA Network Open.*

eTable 4. Human Factors Study Quality Assessment Tool, a Modified Version of the Quality Assessment Informatics Instrument (QUASII)

The QUASII assessment tool, originally developed by Weir et al. (2009),[2] consists of 18 questions, each with a possible score between 1 to 5, and resulting in a maximum study quality score of 90. The modifications to the questions (for use for technology and clinicians), and anchor statements are underlined.

Questions	Anchor Definition					Further clarification
**SECTION ONE**	**Min (1)**	**Mid-low (2)**	**Mid (3)**	**Mid-high (4)**	**Max (5)**	
1. What is your estimate of the overall degree of research quality? (used for validation purposes)	Completely inadequate	Largely inadequate, significant issues	Somewhat adequate	Mostly adequate, few/minor issues	Completely adequate	
2. To what degree does the manipulation and/or measurement of the Independent Variable(s) reflect the underlying construct that was proposed by the authors?	Completely inadequate	Largely inadequate, significant issues	Somewhat adequate	Mostly adequate, few/minor issues	Completely adequate	
3. To what degree was the technology implementation sufficient?	Non-functional or significantly compromised implementation, e.g. paper prototype (Completely inadequate)	Low-fidelity simulation, unrepresentative setting (Largely inadequate, significant issues)	Low-fidelity simulation, representative setting (Somewhat adequate)	High-fidelity simulation, representative setting (Mostly adequate, few/minor issues)	Fully-realized technology, implementation *in situ* (Completely adequate)	Focusing on *realism. Fidelity:* Degree of the resemblance between experimental implementation (i.e. implementation in the study) and real-life implementation with respect to form and function

© 2019 Lin YL et al. *JAMA Network Open.*

Questions	Anchor Definition					Further clarification
4. To what degree were the dependent variable(s) valid and clinically significant? (Is the selected dependent variable (DV) appropriate? Is the impact of the technology on the DV large enough to justify changing clinical practice? (To what degree were the dependent variable(s) valid and clinically significant?)	DV does not appropriately operationalize the construct of interest, is not clinically significant (Completely inadequate)	Largely inadequate, significant issues	Somewhat adequate	Mostly adequate, few/minor issues	DV appropriately operationalizes the construct of interest, is clinically significant (Completely adequate)	*Clinical significance*: Would a significant change in the dependent variable as a result of the treatment/intervention justify changing clinical practice?
5. To what degree was the proposed relationship between the independent variable and the dependent variable specified in terms of mediators and moderators? Was there evidence of	Completely inadequate	Largely inadequate, significant issues	Somewhat adequate	Mostly adequate, few/minor issues	Completely adequate	i.e. is the proposed relationship between the IV and the DV well defined? *Mediator:* intrinsic to the causal process, is a third variable that varies with the IV and explains the relationship between the IV and the DV *Moderator:* the direction or magnitude of the relationship

Questions	Anchor Definition					Further clarification
selection bias in terms of measuring the types of effects (e.g. choosing only those outcomes thought to be favorable)?						between the IV and the DV depends on the moderator
**SECTION TWO**	**Min (1)**	**Mid-low (2)**	**Mid (3)**	**Mid-high (4)**	**Max (5)**	**Further clarification**
6. To what degree do deficiencies in design impact the conclusions? (pre/post designs getting the worst scores)	Pre/post, no evidence of group assessment (Strongly invalidates)		Pre/post, group analyses (Somewhat invalidates)		Randomized Control Trial (Very little effect)	Focusing on the *validity* of the conclusions.
7. To what degree do differences in the type of clinicians between study groups impact the conclusions?	Subjects in each group fundamentally differ, cannot be compared (Strongly invalidates)		Subjects in each group differ somewhat, comparison moderately impacts conclusion validity (Somewhat invalidates)		Subjects in both groups are identical, fully comparable (Very little effect)	Focusing on the *validity* of the conclusions.
8. To what degree do differences in the technology implementation between study groups impact the conclusions?	Significant differences in implementation preclude group comparison (Strongly invalidates)		Minor differences in implementation hinder group comparison (Somewhat invalidates)		Technological implementation is identical, comparable (Very little effect)	Focusing on the *validity* of the conclusions.

© 2019 Lin YL et al. *JAMA Network Open.*

Questions	Anchor Definition					Further clarification
9. To what degree do differences in the way that groups were treated during the study period impact the conclusions?	Significantly different treatment, clear impact on conclusion validity (Strongly invalidates)		Treatment differs somewhat, limited impact on conclusion validity (Somewhat invalidates)		Identical treatment, no influence on conclusion validity (Very little effect)	*Examples of issues that can arise for studies with a control group and a treatment group:*<br>- Compensatory rivalry (when control subjects compare themselves with those treated and are motivated to do as well)<br>- Resentful demoralization (when controls compare themselves to those treated and feel so helpless or deprived that they do poorly)
10. To what degree do differences in the way that measurements were taken during the study period impact the conclusions?	Inconsistent method of measurement, significant impact on conclusion validity (Strongly invalidates)		Somewhat invalidates		Consistent method of measurement, no impact on conclusion validity (Very little effect)	
11. To what degree did the measurement of the dependent variables impact the conclusions? (reliability, validity,	Inappropriate or insufficient measurement, construct of interest is underrepresented (Strongly invalidates)		Somewhat invalidates		Appropriate and sufficient measurement, construct of interest is fully represented (Very little effect)	*Does the measurement procedure used affect construct validity?*<br>- the use of a single measure for a given construct presents a threat to construct validity,

© 2019 Lin YL et al. *JAMA Network Open.*

Questions	Anchor Definition					Further clarification
floor and ceiling effects)						potentially underrepresenting the construct being measured - is there a clear link between the construct of interest and the measures used to operationalize it (i.e. measure it)?
12. To what degree did inappropriate unit of analysis impact the conclusions? (e.g. using a patient level analysis when provider behavior was the target)	Inappropriate unit of analysis, e.g. patient when target is provider behaviour (Strongly invalidates)		Appropriate but non-ideal unit of analysis (Somewhat invalidates)		Fully appropriate, ideal unit of analysis (Very little effect)	*Unit of analysis:* the major entity that is being analyzed in a study. It is the 'what' or 'who' that is being studied.
13. To what degree did the way that confounders were included in the statistical analysis impact the conclusions?	Confounders were not acknowledged or accounted for (Strongly invalidates)		Confounders were identified but inappropriately accounted for (Somewhat invalidates)		No confounders OR confounders were identified and appropriately accounted for (Very little effect)	*Identifying confounders:* Is there academic evidence that the extraneous variables you can identify affect the dependent variable? Are there any logical or practical reasons to assume that an extraneous variable might become a confounding variable?

© 2019 Lin YL et al. *JAMA Network Open.*

Questions	Anchor Definition					Further clarification
14. To what degree did possible problems with missing data impact the conclusions?	Incompleteness of data is explicit, significant impact on conclusions (Strongly invalidates)		Completeness of data is not explicit but no evidence of missing data effect (Somewhat invalidates)		Completeness of data is explicit, no impact on conclusions (Very little effect)	
15. To what degree did the type of statistical analysis done impact the conclusions?	Inappropriate test applied, test conditions violated, or no statistical analysis performed; significant effect on validity of conclusions (Strongly invalidates)		Issues with statistical analysis but limited impact on validity of conclusions (Somewhat invalidates)		Appropriate test applied, test conditions met, no impact on validity of conclusions (Very little effect)	Includes violating assumptions of tests (e.g. violated normality assumption, homogeneity, etc.)
16. To what degree did "fishing" or conducting multiple tests impact the conclusions?	Multiple tests applied; tests do not align with original hypothesis; significant impact on conclusion validity (Strongly invalidates)		Data fishing suspected, some impact on conclusion validity (Somewhat invalidates)		Limited number of tests applied; tests align with original hypothesis; no impact on conclusion validity (Very little effect)	*Data fishing*: Searching for data to fit preconceived ideas; the inappropriate search for statistically significant relationships.<br><br>Performing multiple analyses without adjusting the significance level or using sequential designs.
17. To what degree are the study results generalizable?	Results are not robust, cannot be generalized (Strongly invalidates)		Results are robust, can be generalized to some extent (Somewhat invalidates)		Results are robust, can be generalized to a great extent (Very little effect)	Can the findings be generalized to a wider population, or across populations, treatments, contexts/settings, or time, etc.?

© 2019 Lin YL et al. *JAMA Network Open.*

Questions	Anchor Definition					Further clarification
						To assess the extent to which generalisations can be made: must consider how well the sample (or population) represents the wider population (or settings/contexts, treatments or time) we are interested in making generalisations to.
18. Do the conclusions match the results reported?	Results do not support conclusions (Strongly invalidates)		Results somewhat support conclusions (Somewhat invalidates)		Results fully support conclusions (Very little effect)	

© 2019 Lin YL et al. *JAMA Network Open.*

eReferences

1. Peute LW, Driest KF, Marcilly R, Bras Da Costa S, Beuscart-Zephir MC, Jaspers MW. A framework for reporting on human factor/usability studies of health information technologies. Stud Health Technol Inform 2013;194:54-60.
2. Weir CR, Staggers N, Phansalkar S. The state of the evidence for computerized provider order entry: a systematic review and analysis of the quality of the literature. International journal of medical informatics 2009;78(6):365-374.

© 2019 Lin YL et al. *JAMA Network Open.*
